# Hellebrigenin induces oral cancer cell apoptosis by modulating MAPK signalling and XIAP expression

**DOI:** 10.1111/jcmm.18071

**Published:** 2023-12-03

**Authors:** Ming‐Ju Hsieh, Chia‐Chieh Lin, Yu‐Sheng Lo, Hsin‐Yu Ho, Yi‐Ching Chuang, Mu‐Kuan Chen

**Affiliations:** ^1^ Oral Cancer Research Center Changhua Christian Hospital Changhua Taiwan; ^2^ Doctoral Program in Tissue Engineering and Regenerative Medicine, College of Medicine National Chung Hsing University Taichung Taiwan; ^3^ Graduate Institute of Biomedical Sciences China Medical University Taichung Taiwan; ^4^ Department of Post‐Baccalaureate Medicine, College of Medicine National Chung Hsing University Taichung Taiwan; ^5^ Department of Otorhinolaryngology, Head and Neck Surgery Changhua Christian Hospital Changhua Taiwan

**Keywords:** apoptosis, hellebrigenin, oral squamous cell carcinoma, XIAP

## Abstract

Oral squamous cell carcinoma (OSCC), which accounts for 90% of all oral cancers, has become a public health crisis worldwide. despite advances in therapeutic interventions, the prognosis remains poor for advanced‐stage OSCC. In this study, we investigate the anticancer activity and the mode of action of hellebrigenin in human OSCC. The findings demonstrated that hellebrigenin exerted cytotoxic effects in OSCC cells through cell cycle arrest at the G2/M phase and downregulation of cell cycle‐related proteins (cyclins A2, B1 and D3, Cdc2, CDK4 and CDK6). Moreover, hellebrigenin caused activation of PARP and caspase 3, 8 and 9, followed by downregulation of antiapoptotic proteins (Bcl‐2 and Bcl‐xL) and upregulation of pro‐apoptotic proteins (Bax and Bak). The hellebrigenin treatment also increased Fas, DR5, DcR2 and DcR3 expressions in oral cancer cells, indicating the compound causes oral cancer cell apoptosis through both intrinsic and extrinsic pathways. Regarding upstream signalling, hellebrigenin was found to reduce the phosphorylation of ERK, p38, and JNK, indicating that hellebrigenin triggers caspase‐mediated apoptosis by downregulating MAPK signalling pathway. Finally, the human apoptosis array findings revealed that hellebrigenin specifically suppressed the expression of XIAP to execute its pro‐apoptotic activities. Taken together, the study suggests that hellebrigenin can act as a potent anticancer compound in human OSCC.

## 1 INTRODUCTION

Cancers developed in the oral cavity mucosa are collected termed as oral squamous cell carcinoma (OSCC). It is highly prevalent in developing countries and accounts for 90% of all oral cancers.^1^ Smoking or chewing tobacco and excessive consumption of alcohol are considered to be the most common risk factors for OSCC.^2^ With 377,713 new cases and 177,757 deaths recorded in 2020, oral cancer has become a concerning public health issue worldwide.^3^ Early detection via oral cavity screening and prevention are the major steps to be followed to reduce OSCC‐related morbidity and mortality. However, these types of cancers are often detected in late stages, which makes therapeutic interventions challenging and often unsuccessful.^4^ Advanced‐stage OSCC has a 5‐year survival rate of only 50%.^5^

Surgery together with chemotherapy or radiotherapy is widely used therapeutic intervention for OSCC. While endoscopic removal is possible for early stage tumours, surgery, chemotherapy and radiation, either separately or in combination, are required for locally advanced or metastatic tumours.^6^ Despite the advance in therapeutic strategies, OSCC has a poor prognosis because of its tendency for local recurrence and distant metastasis.^5^ Cisplatin remains the first‐line chemotherapeutic agent for the management of OSCC. It induces cancer cell death by directly damaging DNA and inhibiting DNA replication.^7^ However, cisplatin‐based treatment is associated with severe side effects and the development of drug resistance. Several cisplatin analogues have been developed in order to reduce toxic side effects; however, these analogues have shown significantly lower efficacy than cisplatin at the same dose.^8^ Regarding targeted therapy, cetuximab is the only approved monoclonal antibody for the management of locally advanced or metastatic OSCC. This antibody prevents cancer cell growth by inhibiting epidermal growth factor receptor (EGFR). However, treatment with cetuximab could lead to severe infusion reactions, including acne‐like rash, neutropenia, neuropathy, hypophosphatemia, gastrointestinal tract haemorrhage and liver toxicity.^9^

Biologically active organic compounds, such as cardiotonic steroids, derived from natural resources have shown significant efficacy in treating various health conditions, including infectious diseases, inflammatory diseases, and cancers.^10^, ^11^ Bufadienolides are one kind of cardiotonic steroids and the major effective constituents of cinobufacini, which is a widely used Chinese medicine derived from the dried skin and parotid venom glands of Chinese toad Bufo bufo gargarizans Cantor.^12^ Bufadienolides, which are the major anticancer compounds in cinobufacini, have been shown to reduce the growth of a range of cancer cells, including gastric and hepatocellular cancer cells.^12^, ^13^ Hellebrigenin is an active bufadienolide compound with cytotoxic properties against cancer cells.^14^ Anticancer activities of compound have been observed in a range of malignancies, including colon, lung, breast, pancreatic, prostate, liver and brain cancers.^15^ However, the cytotoxic effect of hellebrigenin has not yet been investigated yet in OSCC. The present study aimed to determine the cytotoxic effect of hellebrigenin in human OSCC. The anticancer mode of action of the compound was also determined in the study.

## 2 MATERIALS AND METHODS

### 2.1 Cell culture and culture conditions

Two human OSCC cell lines, SCC‐1 and SCC‐47, were selected for the experiments. The SCC‐1 cell line was derived from a tumour located on the floor of the mouth of a male patient. The SCC‐47 cell line was derived from the primary tumour of the lateral tongue of a male patient. Both cell lines were obtained from Merck KGaA (Ann Arbor, MI, USA). The Dulbecco's modified Eagle's medium (DMEM; Life Technologies, Grand Island, NY, USA) containing 1% nonessential amino acids (NEAA; Gibco, MA, USA) and 10% fetal bovine serum (Merck Millipore, MA) was used to culture the cells. Human gingival epithelioid S‐G cell line (normal cell lines) was culture in DMEM containing 10% fetal bovine serum. All cells were maintained at 37°C in a humidified atmosphere of 5% CO_2_.

### 2.2 Hellebrigenin treatments

Hellebrigenin (≧98% purity) was purchased from ChemFaces (CheCheng Rd, Wuhan, PRC). The hellebrigenin stock solution (100 μM) was prepared using dimethyl sulfoxide (DMSO) and kept at −20°C for further use. According to the treatment doses, the stock solution was serially diluted to prepare working solutions. The final concentration of DMSO for all treatments was kept at less than 0.1%.

### 2.3 Cell viability assay

MTT (5 mg/mL) assay was used to assess in vitro cytotoxicity of hellebrigenin in SCC‐1 and SCC‐47 cell lines. The cells were cultured in 96‐well plates at 1 × 10^4^ cells/well concentration overnight, followed by treatment with different concentrations of hellebrigenin (0, 2, 4, 8 nM) of 24, 48 and 72 h. The treated cells were incubated with MTT solution at 37°C for 4 h, and the blue formazan crystals formed were dissolved in DMSO. The absorbance was measured at a wavelength of 595 nm using a microplate auto‐reader (BioTek, Winooski, VT, USA).

### 2.4 Colony formation assay

The SCC‐1 and SCC‐47 cell lines were seeded onto 6‐well plates at 5 × 10^3^ cells/well concentration. Afterwards, the cells were treated with different concentrations of hellebrigenin (0, 2, 4, 8 μM) and cultured for 10 days, with media changing every 3 days. The cells were then fixed with methanol and the colonies were stained with 10% Giesa (Merck Sigma) stain solution diluted with water. Afterwards, the colonies were photographed and counted for record.

### 2.5 Cell cycle analysis

The SCC‐1 and SCC‐47 cell lines were seeded onto 6‐well plates with appropriate cell numbers. After 24 h of treatment with various concentrations of hellebrigenin (0, 2, 4, 8 nM), the cells were fixed in 70% ethanol for 16–20 h at −20°C. The frozen cells were then incubated with propidium iodide (PI) staining solution (BD Biosciences, Franklin Lakes, NJ, USA) for 30 min in the dark at room temperature. Subsequently, the data were analysed using BD CSampler Plus software (version 10; BD Biosciences).

### 2.6 Annexin V/PI double staining assay

The SCC‐1 and SCC‐47 cell lines were seeded onto a 6‐well plate at a density of cells 3 × 10^5^ per well and treated with hellebrigenin (0, 2, 4, 8 nM) for 24 h. Then, the cells were incubated with both PI solution (BD Biosciences) and annexin V‐fluorescein isothiocyanate solution for 20 min in the dark at room temperature. The data were analysed using BD CSampler Plus software (version 10; BD Biosciences).

### 2.7 Mitochondrial membrane potential analysis

The SCC‐1 and SCC‐47 cell lines were seeded onto a 6‐well plate at a density of 3 × 10^5^ per well and treated with hellebrigenin (0, 2, 4 and 8 nM) for 24 h. The cells were then stained with Mitochondrial Membrane Potential detection JC‐1 kit working solution (BD Bioscirnces). The working solution reacted for 10–15 min at 37°C in a CO_2_ incubator, and the cells were analysed by flow cytometry. The data were analysed using BD CSampler Plus software (version 10; BD Biosciences).

### 2.8 DAPI Staining

The SCC‐1 and SCC‐47 cell lines were cultured onto a 6‐well plate and treated with hellebrigenin (0, 2, 4 and 8 nM). The cells were fixed with 4% formaldehyde for 30 min at room temperature. The DAPI reagent (50 μg/mL) was dissolved (1:10,000) in triton‐X100 mixed with 1 × PBS solution and incubated with the cells for 15 min. The cells were then observed under fluorescence microscopy (Lecia, Bensheim, Germany) and photographs were taken.

### 2.9 Western blotting analysis

The SCC‐1 and SCC‐47 cell lines were seeded onto 6 cm dishes with 5 × 10^5^ cells per dish and treated with different concentrations of hellebrigenin (0, 2, 4 and 8 nM) for 24 h. Next, the RIPA cell lysis buffer containing protease/phosphatase inhibitor cocktails (EMD Millipore) was added to the cells to extract protein. After quantification with BCA Cell Quantification (Thermo Fisher Scientific) assay, the protein samples were mixed with dye (1:4) and separated on 12.5% and 15% polyacrylamide gel, followed by transfer onto a 0.22 mm PVDF membrane (EMD Millipore). Afterwards, the membrane was blocked with 5% skim milk for 1 h at room temperature and incubated with the indicated primary antibodies overnight at 4°C. The next day, appropriate secondary antibodies were added to the membrane, and the protein bands were visualized with chemiluminescence fluorescence Image Quant LAS 4000 (GE Healthcare, Berlin, Germany) biomolecule imaging system.

### 2.10 Proteome profiler analysis

The SCC‐1 and SCC‐47 cells were lysed using RIPA lysis buffer. The whole cell lysates were injected into Proteome Profiler Human Apoptosis Array kit (R&D Systems Inc., cat. ARY009; Minneapolis, MN, USA). Capture antibodies were spotted in duplicate on the nitrocellulose membrane. Target analytes were detected using a biotinylated or horseradish peroxidase (HRP)‐conjugated detection antibody and measured by ImageQuant LAS 4000 Mini (GE Healthcare Life Sciences).

### 2.11 RNA interference experiments

The SCC‐1 and SCC‐47 cell lines were seeded onto 6‐well plates with appropriate cell numbers. The Human XIAP siRNA (Cohesion Biosciences, London, UK) and Negative control were transfected with Turbofect reagent (Thermo Fisher Scientific; Waltham, MA, USA) dissoved in a DMEM medium (FBS free). Next day, the transfected cells were treated with hellebrigenin (8 nM) for 24 h. The protein samples extracted from the cells were further detected by Western blot analysis.

### 2.12 In vivo antitumor growth effects of hellebrigenin on xenograft transplantation

As previously described,^16^ for the experimental study of xenograft growth inhibition, 5–6‐week‐old male C57BL/6 mice (18–22 g) (National Taiwan University Animal Center, Taiwan) were used. SCC‐1 cells (2 × 10^6^ per mouse) were resuspended in 100 μL of sterile PBS and injected sc into the right flank of the mouse. Mice were randomized into two groups (4 mice/group). All animals were housed with a regular 12‐h light/12‐h dark cycle and ad libitum access to a standard rodent food diet (LaboratoryRodent Diet 5001, LabDiet, St. Louis, MO) and kept in a pathogen‐free environment in the Laboratory Animal Unit (temperature 22°C, humidity 30–70%, 5 mice/cage). Seven days after tumour cell injection, mice were fed hellebrigenin (6 mg/kg) three times a week. The control group received an equal volume of 0.5% carboxymethylcellulose vehicle. Tumour volumes were determined from calliper measurements obtained every 3 days. At the end of the experiment, the mice were sacrificed and the primary tumours were removed for further analysis. Primary tumours were separated from the surrounding muscles and dermis and then weighed. Tumour volume was calculated using the following formula: 0.5 × length × width.^2^ The mean weight of the mice at the start of the study and at the end of the study did not differ significantly between the groups. All animal procedures were conducted according to the institutional animal welfare guidelines of the Institutional Animal Care and Use Committee (IACUC) of Changhua Christian Hospital.

### 2.13 Statistical analysis

Statistical analyses were performed using GraphPad Prism Software Version 5.0 (GraphPad Software Inc., La Jolla, CA). Each experiment was replicated at least three times. One‐way anova with Tukey's multiple comparisons test was used to compare the differences between two groups (control and drug dose). The quantitative data are expressed as the mean ± standard deviation (SD). A *p* value of <0.05 was considered statistically significant.

## 3 RESULTS

### 3.1 Effects of hellebrigenin on the viability of oral cancer cells

**FIGURE 1:**
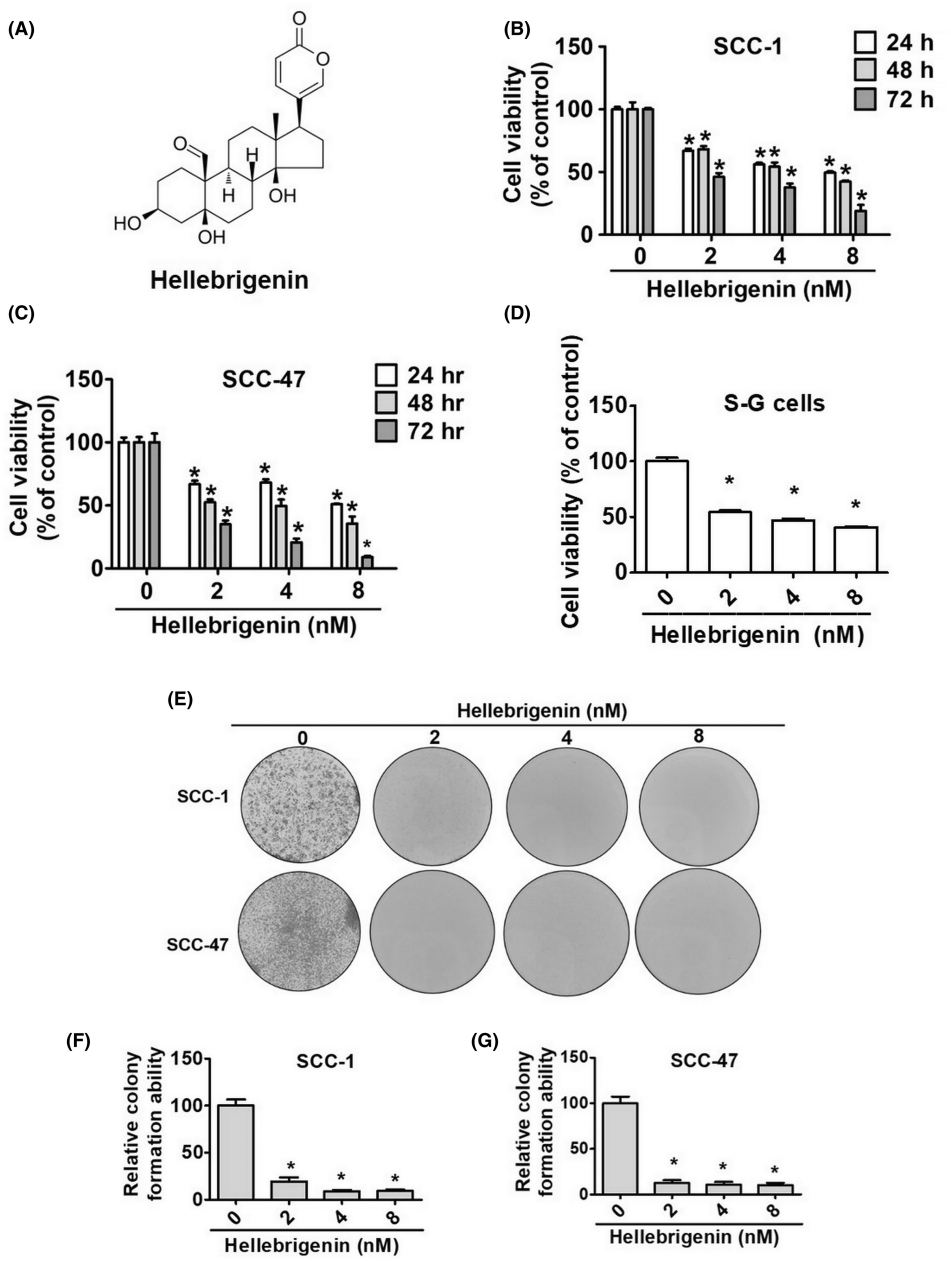
Cytotoxicity effect and cell proliferation of hellebrigenin induces in different oral cancer cells. (A) Chemical structure of hellebrigenin. The cell viability of MTT assay in SCC‐1 (B), SCC‐47 (C) and (D) S‐G cell line with different concentrations (0, 2, 4 and 8 nM) of hellebrigenin for 24, 48 and 72 h. (E) After hellebrigenin treatment of SCC‐1 and SCC‐47, cells were confirmed by *colony* formation assay culture for 10 days. Quantitative analysis of *number of colonies* for SCC‐1 (F) and SCC‐47 (G) cells. All data are expressed as mean ± SD of three independent experiments. *p < 0.05, compared with the control group.

To investigate the cytotoxic effects of hellebrigenin in oral cancer cells, we treated two OSCC cell lines (SCC‐1 and SCC‐47) with different hellebrigenin concentrations (2, 4 and 8 nM) at different timepoints and conducted MTT and colony formation assay. Cells without any treatment served as experimental controls. As observed in Figure 1B–D, the hellebrigenin treatment significantly reduced oral cancer cell and normal cell lines (human gingival epithelioid S‐G cell line) viability in a dose‐ and time‐dependent manner. Similarly, we found that all tested doses of hellebrigenin prevented OSCC colony formation (Figure 1E–G). Overall, the results highlight the cytotoxic activity of hellebrigenin in OSCC.

### 3.2 Effects of hellebrigenin on cell cycle progression and cell cycle‐related protein expression in oral cancer cells

**FIGURE 2:**
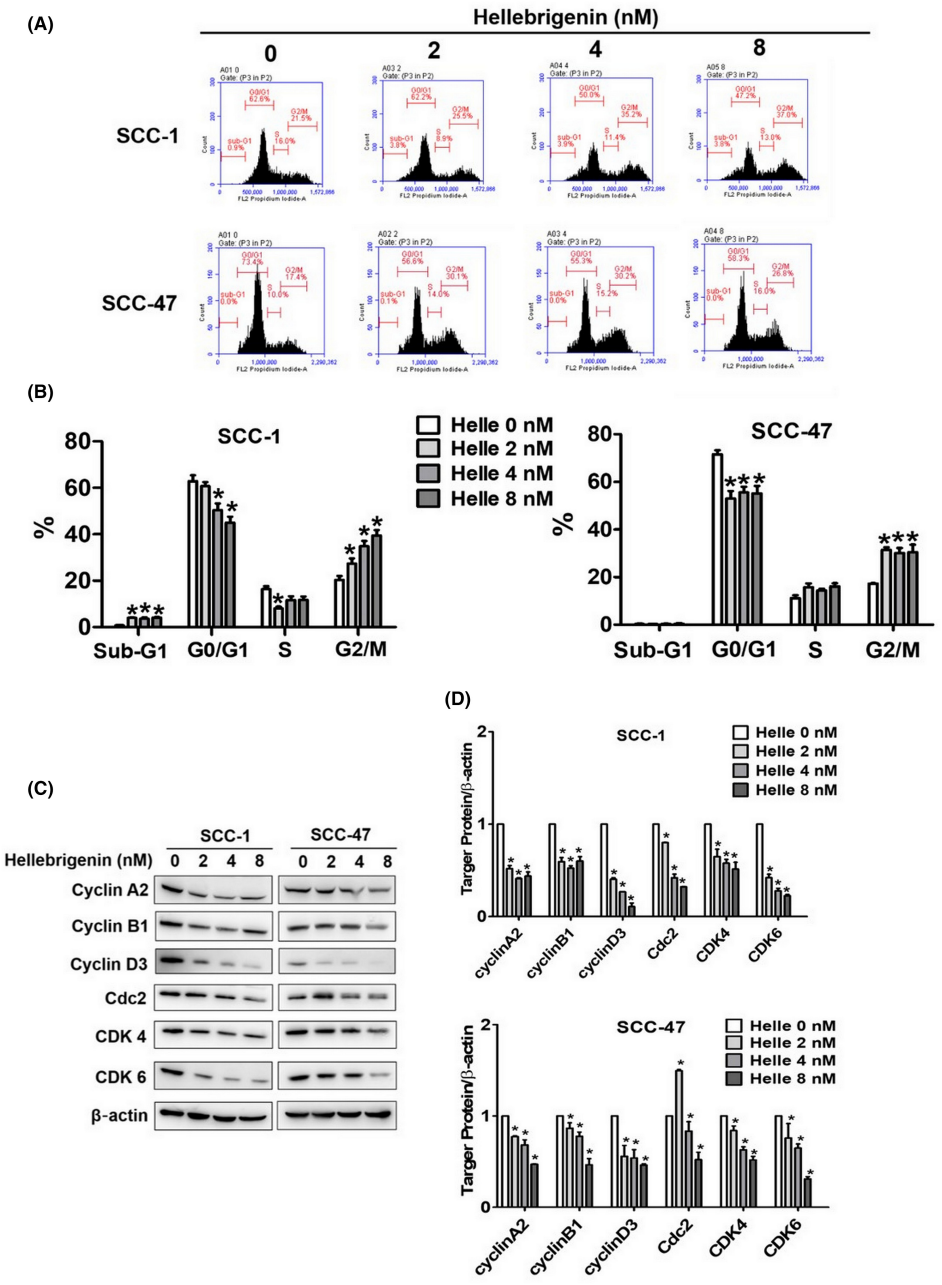
Effect of hellebrigenin on cell cycle progression and cell cycle‐related protein in oral cancer cells. (A) Hellebrigenin caused SCC‐1 and SCC‐47 cell cycle arrest in the G2/M phases (B) The quantitative analysis of cell cycle about sub‐G1, G0/G1, S, G2/M for SCC‐1 and SCC‐47 cell lines. (C) Western blot of Cyclin A2, Cyclin B1, Cyclin D3, Cdc2, CDK4, CDK6 protein expression of SCC‐1 and SCC‐47 cells. (D) Quantitative analysis of two oral cancer cell protein expression being adjusted with β‐actin. All data are expressed as mean ± SD of three independent experiments. *p < 0.05, compared with the control group.

To investigate whether hellebrigenin exerts cytotoxic effects by affecting cell cycle progression, we treated cancer cells with different hellebrigenin concentrations and conducted cell cycle analysis. In addition, we extracted protein samples from treated cells and checked the expressions of various cell cycle regulatory proteins (cyclins A2, B1 and D3, Cdc2, CDK4 and CDK6). As seen in Figure 2A,B, the hellebrigenin treatment caused cell cycle arrest at the G2/M phase in both OSCC cell lines. In addition, the hellebrigenin treatment significantly and dose‐dependently reduced the expressions of cell cycle regulatory proteins (Figure 2C,D). Overall, these findings indicate that hellebrigenin prevents OSCC proliferation by inhibiting cell cycle progression.

### 3.3 Effect of hellebrigenin on apoptotic pathway in oral cancer cells

**FIGURE 3:**
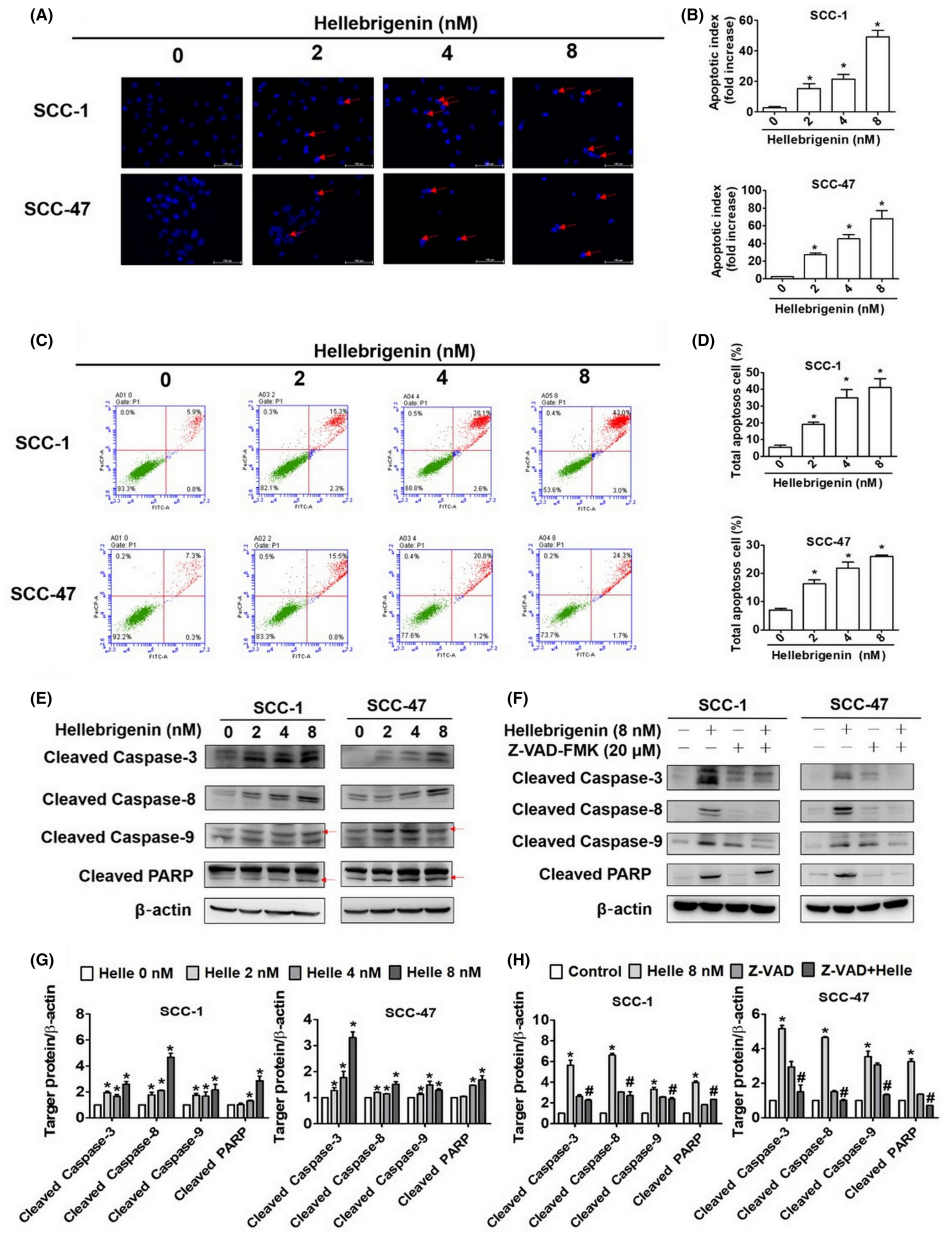
Hellebrigenin induces cell apoptosis and caspase induction in oral cancer cells. (A) Analysis of early phase of intrinsic apoptosis by DAPI staining of SCC‐1 and SCC‐47 cells via fluorescence microscopy. (B) The quantitative analysis of DAPI staining of different oral cancer cell lines. (C) Two oral cancer cell lines were treated with 0, 2, 4 and 8 nM hellebrigenin, analysed through flow cytometry after annexin‐V/PI double staining and (D) quantified the results of both cells. (E) The protein expression of cleaved caspase‐3, cleaved caspase‐8, cleaved caspase‐9 (red arrows) and cleaved PARP (red arrows) proteins were analysed through Western blotting. (F) Caspase inhibitor Z‐VAD‐FMK was combined with hellebrigenin and detected apoptosis‐related protein. (G, H) Quantitative analysis of two oral cancer cell protein expressions being adjusted with β‐actin. All data are expressed as mean ± SD of three independent experiments. *p < 0.05, compared with the control group.

**FIGURE 4:**
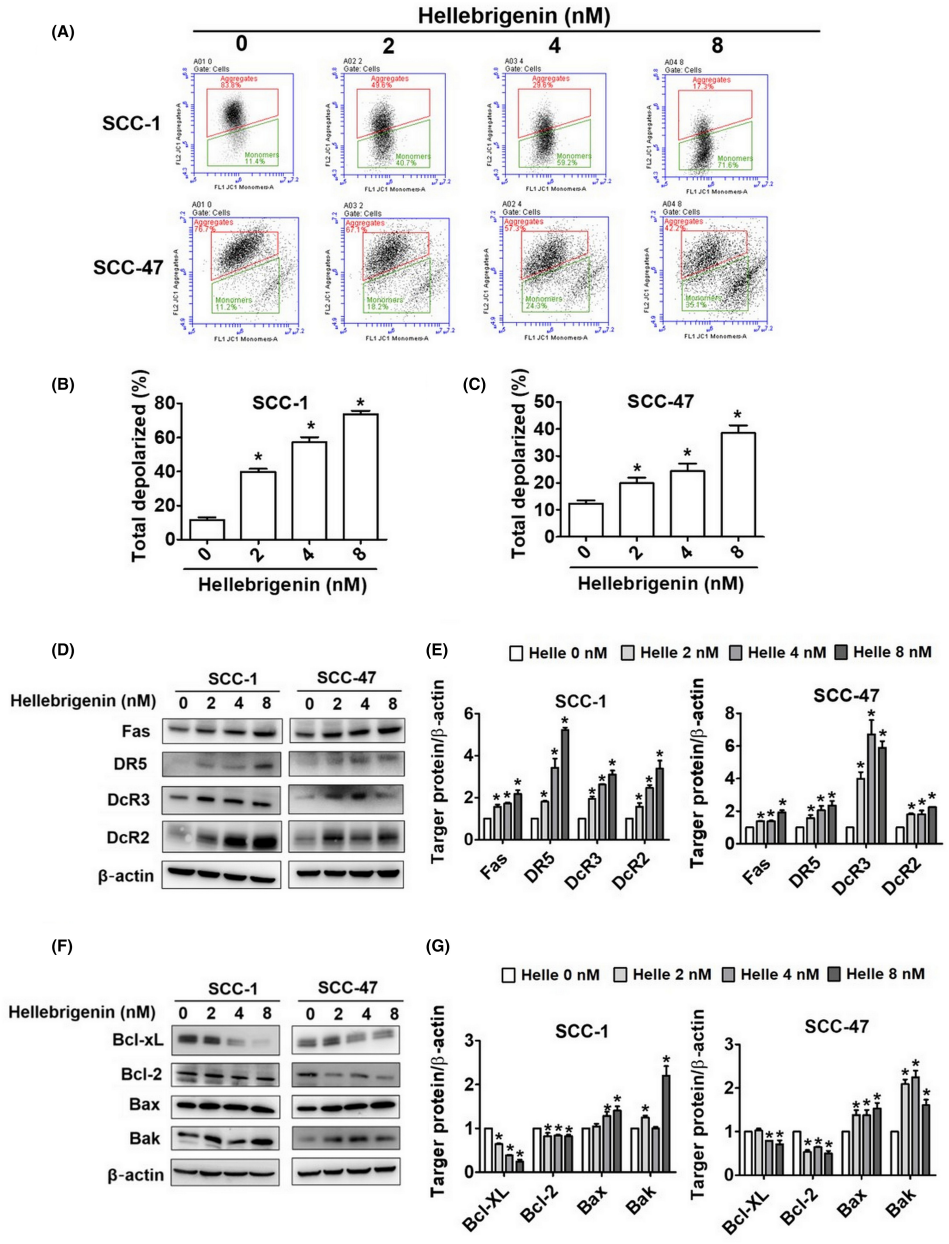
Hellebrigenin activates apoptosis via the mitochondrial and death receptor pathways in oral cancer cells. (A) SCC‐1 and SCC‐47 cell lines were treated with different concentrations of hellebrigenin (0–8 nM) to analyse JC‐1 dye for mitochondrial membrane potential through flow cytometry. Total depolarized percentage of SCC‐1 (B) and SCC‐47 (C) are shown. After treatment of hellebrigenin on SCC‐1 and SCC‐47 cells, (D, E) Western blotting detected the expression change in Fas, DR5, DcR3, DcR2 and quantitative data of protein expression after being adjusted with β‐actin. (F, G) The antiapoptotic proteins (Bcl‐xL and Bcl‐2) and proapoptotic (Bax and Bak) were measured and definite quantitative determination after Hellebrigenin treatment. All data are expressed as mean ± SD of three independent experiments. *p < 0.05, compared with the control group.

To further determine the mode of action of hellebrigenin, we calculated the apoptotic index by assessing chromatin condensation and DNA fragmentation in treated cells. As seen in Figure 3A,B, the hellebrigenin treatment dose‐dependently elevated the apoptotic index. Next, we conducted flow cytometry to measure the number of dead cells. The results indicated that hellebrigenin dose‐dependently increased the percentage of apoptotic cells in both cell lines (Figure 3C,D). In accordance with these findings, we observed activation of caspases 3, 8 and 9 and PARP (Figure 3E,G). Furthermore, we cotreated the cells with hellebrigenin and a pan‐caspase inhibitor (Z‐VAD‐FMK) and observed that the cotreatment significantly mitigated the effect of hellebrigenin on caspase and PARP activation (Figure 3F,H). Overall, the results demonstrate that hellebrigenin causes OSCC cell death by activating caspases and PARP. By analysing specific apoptotic mechanisms, we observed that the hellebrigenin treatment altered mitochondrial membrane potential (Figure 4A,B). We next assessed the expressions of proteins related to different apoptotic pathways in treated cells. As seen in Figure 4D,E, the hellebrigenin treatment dose‐dependently increased the expressions of FAS, death receptor (DR5) and death decoy receptors (DcR2 and DcR3). Moreover, we observed hellebrigenin‐mediated induction in pro‐apoptotic proteins Bax and Bak and a reduction in anti‐apoptotic proteins Bcl‐2 and Bcl‐xL (Figure 4F,G). Overall, the results demonstrate that hellebrigenin executes oral cancer cell apoptosis by activating both intrinsic and extrinsic pathways.

### 3.4 Effect of hellebrigenin on MAPK pathway in oral cancer cells

**FIGURE 5:**
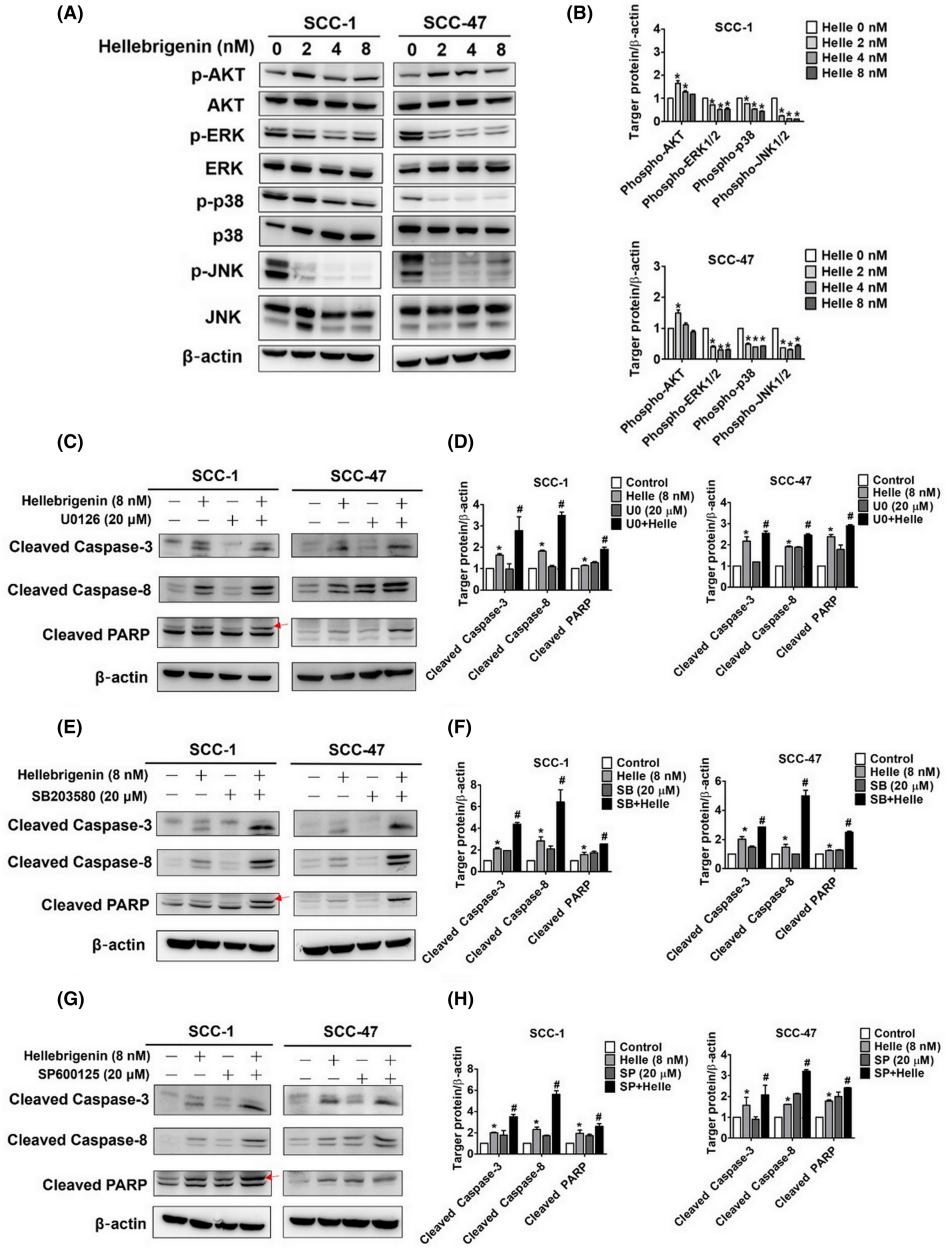
Hellebrigenin‐regulated cell apoptosis through the ERK, p38 and JNK pathways. After being treated with hellebrigenin (0–8 nM) for 24 h, (A, B) SCC‐1 and SCC‐47 cells were performed with Western blot analysis and detected a decreased antibody in phosphorylation of ERK, p38 and JNK and quantified the results of both cells. (C–H) The protein expression of cleaved caspase‐3, cleaved caspase‐8 and cleaved PARP were detected after treatment of U0126 (20 μM), SB203580 (20 μM) or SP600125 (20 μM) for 1 h and co‐treated with hellebrigenin (8 nM) for another 24 h. All quantitative data of protein expression after being adjusted with β‐actin. The experiments were expressed as mean ± SD of three independent experiments. *p < 0.05, compared with the control group.

The MAPK pathway plays a central role in several biological processes, including cell growth, differentiation, development and apoptosis.^17^ In many cancer types, aberrant expressions of proteins related to the MAPK pathway have been observed.^18^ In the present study, we investigated whether hellebrigenin exerts cytotoxic effects by modulating MAPK signalling. Specifically, we assessed the phosphorylation of ERK, p38 and JNK in oral cancer cells treated with different concentrations of hellebrigenin. We also assessed the expression of AKT in these cells. As presented in Figure 5A,B, ERK, p38 and JNK expressions decreased considerably after the hellebrigenin treatment. However, no significant and consistent change in AKT phosphorylation was observed. Given the significant changes in MAPK pathway components, we next treated the cells with hellebrigenin with or without specific ERK (U0126), p38 (SB203580) or JNK inhibitor (SP600125) and determined the activation of PARP and caspases 3, 8 and 9. As shown in Figure 5C,D, the cells treated with both hellebrigenin and ERK inhibitors exhibited higher activation of PARP and caspases 3, 8 and 9 than those treated with hellebrigenin alone. Similar results were obtained in cells cotreated with hellebrigenin and p38 or JNK inhibitor (Figure 5E–H). Taken together, these findings indicate that hellebrigenin induces oral cancer cell apoptosis by downregulating MAPK pathway components.

### 3.5 Effect of hellebrigenin on apoptosis‐related protein expressions in oral cancer cells

**FIGURE 6:**
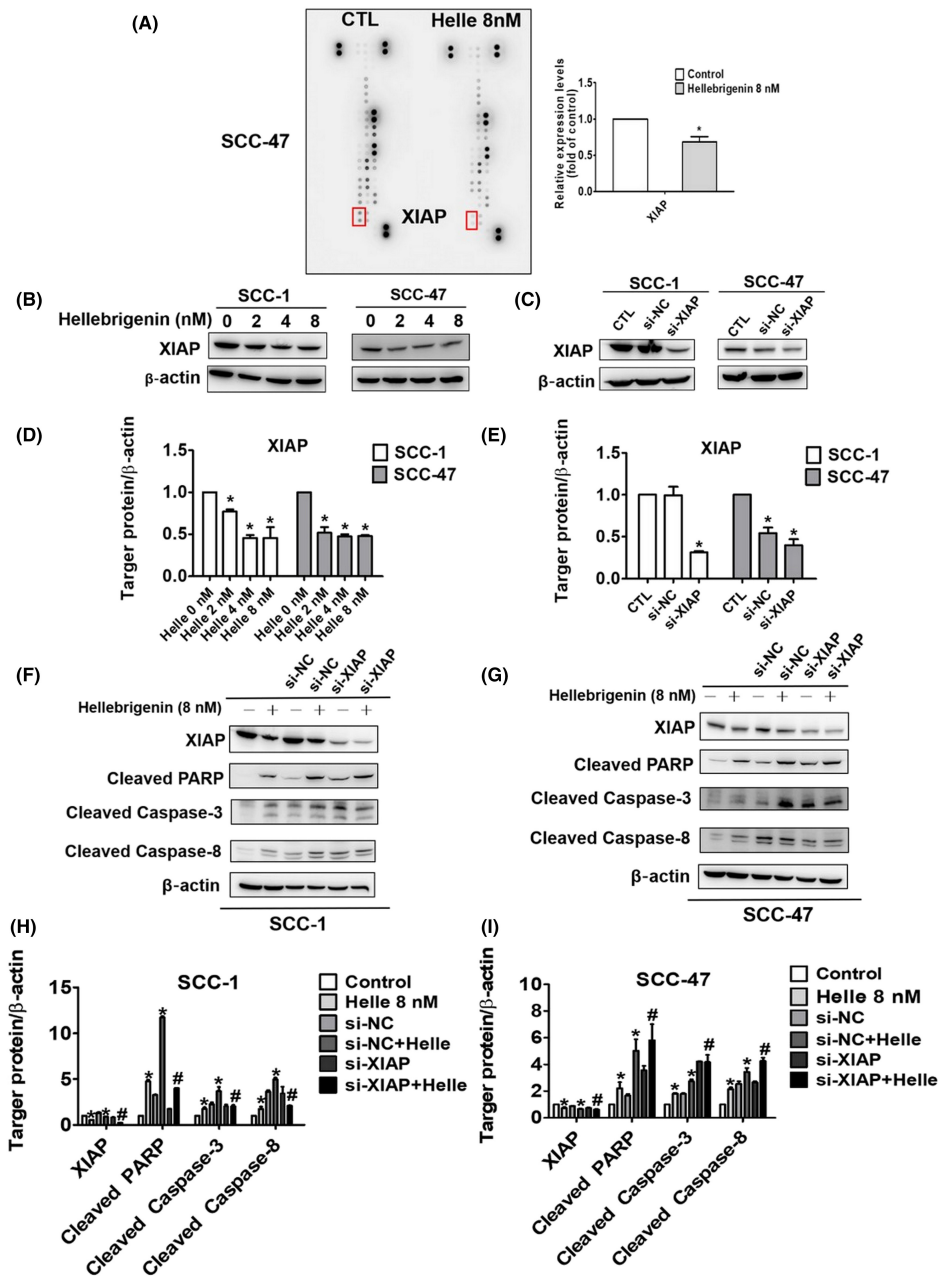
X‐linked inhibitor of apoptosis protein involved in hellebrigenin‐induced caspase activation in oral cancer cells. (A) Representative images of the Human Apoptosis Array (R&D System) are shown of hellebrigenin‐treated after 24 h and quantitative protein expression correction in SCC‐47 cells. (B–E) Western blot analysis was used to detect the expression levels of XIAP after being treated with hellebrigenin (0–8 nM) and transfected with control siRNA or XIAP‐specific siRNA in SCC‐1 and SCC‐47 cells. β‐Actin protein level was used to adjust quantitative results. (F–I) The effect of the combination of XIAP‐specific siRNA and hellebrigenin (8 nM) on the protein level of cleaved PARP, cleaved caspase‐3 and cleaved caspase‐8 was analysed by Western blot assay and *quantitative* data of protein expression after being adjusted with β‐actin. The experiments were expressed as mean ± SD of three independent experiments. *p < 0.05, compared with the control group.

We conducted a human apoptosis array in oral cancer cells to identify apoptosis‐related proteins that are specifically affected by the hellebrigenin treatment. As presented in Figure 6A,B,D, the hellebrigenin reduced the expression of X‐linked inhibitor of apoptosis protein (XIAP) in both cell lines. To further investigate the involvement of XIAP, we knocked down its expression in cells by specific siRNA (Figure 6C,E) and subsequently treated the cells with hellebrigenin (8 nM). As presented in Figure 6F–I, hellebrigenin‐treated XIAP‐knockdown cells exhibited higher PARP and caspases 3, 8 and 9 activations than hellebrigenin‐treated XIAP‐expressing cells. Overall, these findings demonstrate that hellebrigenin initiates oral cancer cell apoptosis by downregulating XIAP expression.

### 3.6 Significant antiproliferative effects of hellebrigenin in the orthotopic graft model

**FIGURE 7:**
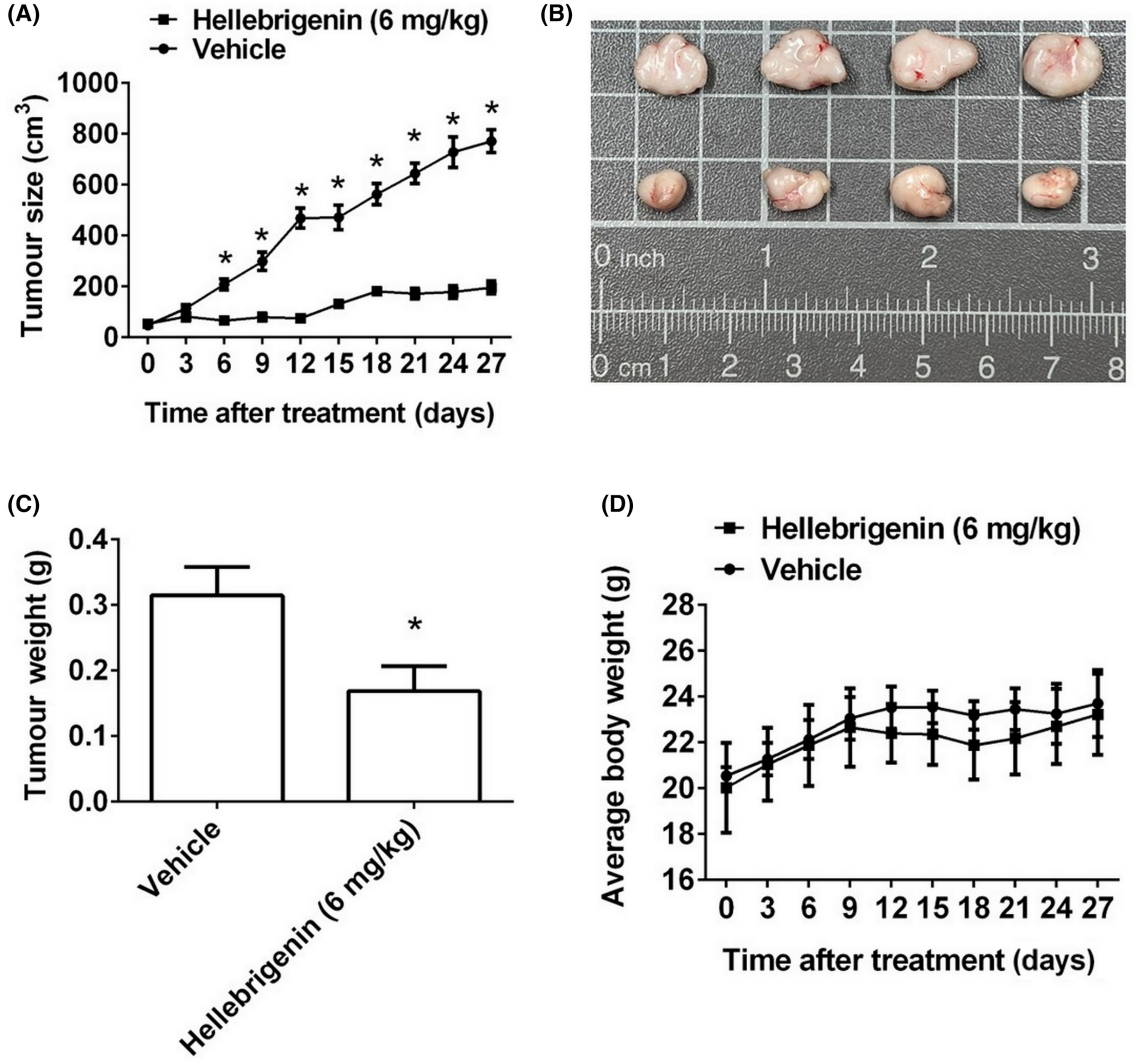
Hellebrigenin suppressed tumour growth of SCC‐1 cells in vivo. (A, B) SCC‐1 cells were injected into the right flank of 6‐week‐old male nude mice. After injection of SCC‐1 cells, nude mice were treated with vehicle or hellebrigenin (6 mg/kg) three times a week. The growth of the xenograft tumours was referred to as the measurement of the long and short dimensions of the tumours, and the calculation of the tumour size was described in Section 2. (C) Tumour weight and (D) body weight changes of the mice during treatment. *p < 0.05, compared to vehicle.

To test the effect of hellebrigenin on tumour growth, the in vivo antitumor effect of hellebrigenin was evaluated. Tumour volumes were determined by measuring the calliper every 3 days. The control group of animals that transplanted SCC‐1 cancer cells showed a progressive increase in their tumour volumes. In mice treated with hellebrigenin receiving 6 mg/kg, mean tumour volume (Figure 7A,B) and tumour weight (Figure 7C) were significantly inhibited compared to vehicles treated. As illustrated in Figure 7D, no significant differences in body weight were detected between these groups. These results showed that hellebrigenin‐treated animals show antitumor growth compared to control animals.

## 4 DISCUSSION

The present study describes the cytotoxic activity and anticancer mechanism of hellebrigenin in human OSCC. Hellebrigenin is one of the major active bufadienolide compounds derived from toad skin secretions and plants of Urginea.^11^ It is a cardiac steroid that acts as an inhibitor of adenosinetriphosphatase.^19^ The anticancer properties of the compound have been demonstrated in a range of cancers, including breast, pancreatic, liver and brain cancers.^14^, ^15^, ^20^, ^21^ However, none of the studies have so far investigated the anticancer effect of hellebrigenin in human OSCC.

In this study, we treated two OSCC cell lines with different doses of hellebrigenin and conducted a series of in vitro experiments to determine the anticancer effect of hellebrigenin. Our results demonstrated that hellebrigenin prevents OSCC proliferation (Figure 1) by inhibiting the cell cycle at the G2/M phase and suppressing expressions of cell cycle regulators (Figure 2). Moreover, the compound induced caspase‐mediated apoptosis through both intrinsic and extrinsic pathways (Figures 3 and 4). Our findings are in line with previous studies investigating the anticancer mode of action of hellebrigenin in different cancer types. Previous studies conducted on oestrogen receptor (ER)‐positive and triple‐negative breast cancer cells have demonstrated that hellebrigenin prevents cancer cell proliferation through cell cycle arrest at the G2/M phase, suppressing cell cycle regulators and inducing caspase‐mediated apoptosis.^21^ Similar findings have been observed in pancreatic and hepatic cancer cells following treatment with hellebrigenin.^15^, ^20^

Previous studies investigating the anticancer mode of action of hellebrigenin have shown that both hellebrin and hellebrigenin bind to and inhibit Na+/K+ ATPase with equal potency, leading to activation of caspase‐mediated apoptosis of cancer cells.^22^, ^23^, ^24^, ^25^ Moreover, hellebrigenin has been found to induce leukaemia cell apoptosis by dysregulating microtubule arrangement.^26^ Hellebrigenin has also been found to induce intracellular reactive oxygen species (ROS) production to trigger cancer cell apoptosis.^27^ In this study, hellebrigenin has also been found to induce intracellular ROS production in SCC‐1 and SCC‐47 cancer cell (Figure S1).

Regarding upstream signalling pathways, we found that hellebrigenin suppressed the phosphorylation of MAPK pathway components ERK, p38 and JNK to induce apoptosis (Figure 5). In accordance, one recent study on cardiac glycosides has revealed that lanatoside induces cell cycle arrest and apoptosis in breast, lung and liver cancer cells by downregulating MAPK signalling.^28^ Ouabain, another cardiac glycoside, has been found to inhibit neutrophil migration by suppressing p38 signalling pathway.^29^ Similarly, bufalin, a cardiotonic steroid, has been found to reduce cancer cell migration/invasion and IL‐1β‐induced proliferation of rheumatoid arthritis fibroblast‐like synoviocytes by downregulating the expression of MAPK signalling components.^30^, ^31^ In contrast to these studies and our observations, one recent study has shown that hellebrigenin treatment cause activation of p38 signalling pathway in glioblastoma cells.^14^ This finding indicates that the mode of action of hellebrigenin is cell type‐specific. However, this study has shown that suppression of p38 signalling by a small molecular inhibitor further potentiate the cytotoxic effect of hellebrigenin in glioblastoma.

Our human apoptosis array findings have highlighted that hellebrigenin induces OSCC cell death by specifically downregulating XIAP (Figure 6), which is a well‐established anti‐apoptotic protein belonging to the inhibitor of apoptosis protein (IAP) family. XIAP acts as an anti‐apoptotic protein by directly neutralizing caspases via its baculovirus‐IAP‐repeat (BIR) domains.^31^ In line with our findings, the XIAP‐downregulating effect of bufotalin has been previously identified in cervical cancer cells.^32^ Similarly, in osteosarcoma cells, cinobufagin has been found to significantly reduce the expression of XIAP.^33^ The whole skin extract of Venenum bufonis has also been shown to cause lung cancer cell death by downregulating XIAP.^34^ Taken together, these findings justify our human apoptotic array findings, which identify XIAP as a specific target of hellebrigenin for the execution of anticancer activity in human OSCC. XIAP, anti‐apoptotic protein and several drugs targeting XIAP, such as Embelin, ASTX660 and Debio1143, are developed and studied for their potential therapeutic use in cancer treatment. Previous study results showed that embelin, an inhibitor of XIAP, exhibited anticancer activity against Ca9‐22 via both autophagy and apoptosis.^35^ A new cIAP1/2 and XIAP antagonist called ASTX660 makes HNSCC tumour cells more susceptible to TNF‐, TRAIL‐ and FasL‐induced cell death. When combined with XRT and PD‐1 blockade in vivo, ASTX660 significantly enhanced delay of tumour growth and rejection rates of established syngeneic murine oral cancer (MOC) 1 tumours.^36^ Phase I Trial of Debio 1143, an antagonist of Inhibitor of apoptosis proteins, which enhances tumour response with concomitant chemoradiotherapy.^37^ The results of these XIAP inhibitors indicate that play an important role in the mechanisms of cancer treatment, whether it is inhibiting cancer cell proliferation or enhancing the sensitivity of chemotherapy drugs. In this study, compared with these XIAP inhibitors, hellebrigenin showed significant advantages in terms of drug dosage. Additionally, hellebrigenin also demonstrated significant anti‐tumour effects in animal in vivo studies, suggesting their potential for cancer research in the future.

## 5 CONCLUSION

The present study describes the anticancer activity of hellebrigenin in human OSCC cells. The study findings reveal that hellebrigenin exerts cytotoxic effects in oral cancer cells by inducing cell cycle arrest and downregulating the expression of a number of cell cycle regulators. Moreover, hellebrigenin causes mitochondrial membrane depolarization and induces caspase‐mediated apoptosis through both intrinsic and extrinsic pathways. The analysis of the MAPK pathway shows that hellebrigenin downregulates the phosphorylation of ERK, p38 and JNK as well as suppresses the expression of XIAP to execute its pro‐apoptotic activities. Overall, the study suggests that hellebrigenin can be used clinically to manage human OSCC.

## AUTHOR CONTRIBUTIONS

**Ming‐Ju Hsieh:** Conceptualization (equal); writing – original draft (lead); writing – review and editing (lead). **Chia‐Chieh Lin:** Methodology (equal); software (equal). **Yu‐Sheng Lo:** Methodology (equal); software (equal). **Hsin‐Yu Ho:** Methodology (equal); software (equal). **Yi‐Ching Chuang:** Methodology (equal); software (equal). **Mu‐Kuan Chen:** Conceptualization (equal).

## CONFLICT OF INTEREST STATEMENT

The authors declare that there are no conflicts of interest.

## Supporting information


Figure S1.

## Data Availability

The data used to support the findings of this study are available from the corresponding author upon request.
