# Supplementary material for: Hellebrigenin induces oral cancer cell apoptosis by modulating MAPK signalling and XIAP expression
Source: J Cell Mol Med. 2023 Dec 3;28(2):e18071. doi: 10.1111/jcmm.18071 (PMC10826427; doi:10.1111/jcmm.18071)
Supplement: Supplementary file 1 — Figure S1. [file JCMM-28-e18071-s001.docx]

**Supplementary Figure 1**


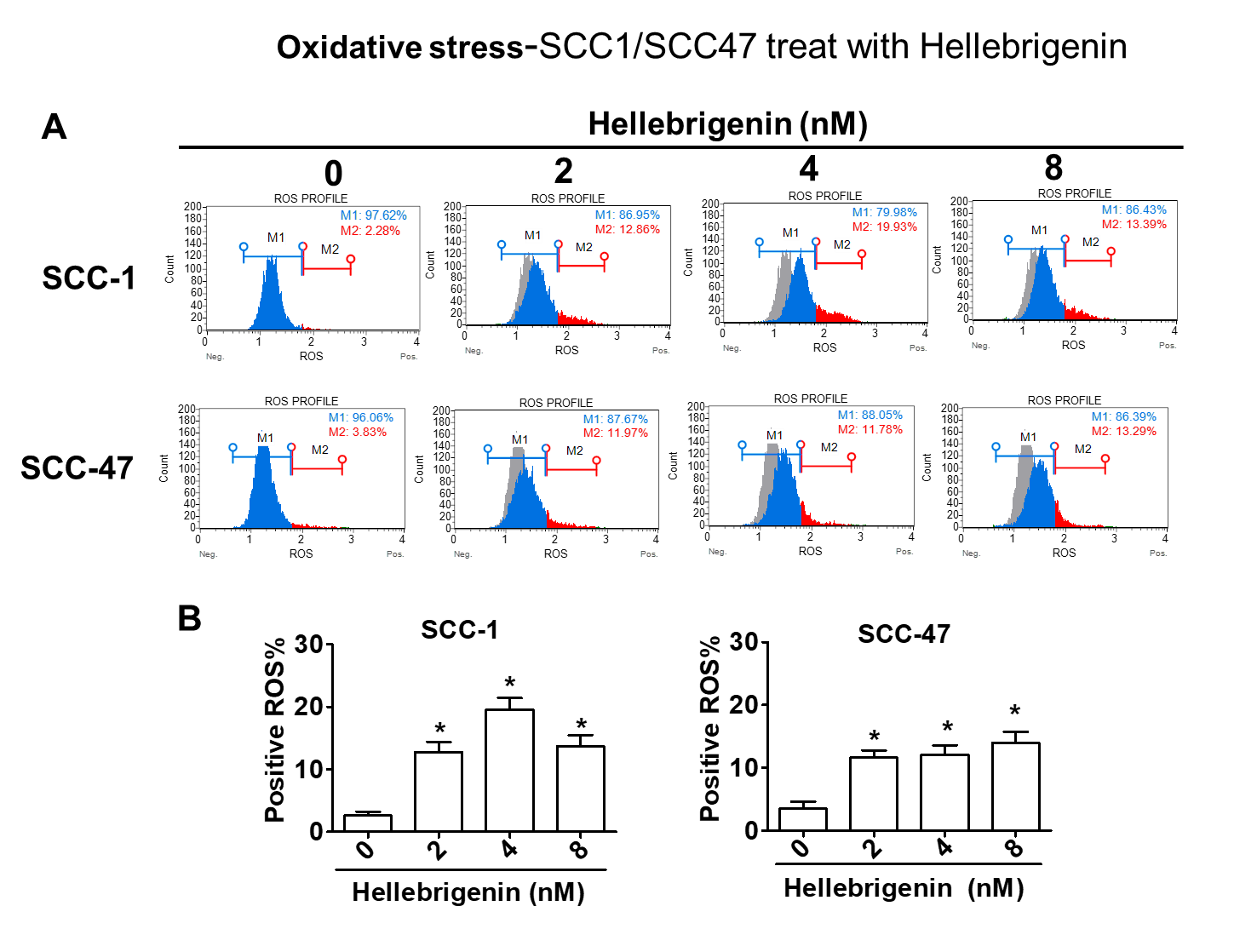


**Figure S1. The reactive oxygen species (ROS) effect of hellebrigenin induces in different oral cancer cells.** (A) Two oral cancer cell lines were treated with 0, 2, 4, 8 nM hellebrigenin and analyzed through flow cytometry by LUMINEX Muse Oxidative Stress Kit, and (B) quantify the results of both cells. All data are expressed as mean ± SD of three independent experiments. *P<0.05, compared with the control group.
